# First records of two mackerel shark species (*Carcharodon planus* comb. nov. and *Carcharodon hubbelli*; Lamnidae) from New Zealand

**DOI:** 10.1080/03036758.2023.2278730

**Published:** 2023-12-05

**Authors:** Dana J. Ehret, Alan J. D. Tennyson, Marcus D. Richards, Robert W. Boessenecker

**Affiliations:** a New Jersey State Museum, Trenton, USA; b Museum of New Zealand Te Papa Tongarewa, Wellington, New Zealand; c Department of Geology, University of Otago, Dunedin, New Zealand; d Palmetto Paleontology Foundation, Summerville, USA

**Keywords:** Lamnidae, Kaipuke Siltstone, Greta Formation, Mount Harris Formation, Miocene

## Abstract

The fossil record of the genus *Carcharodon* (Lamniformes: Lamnidae) dates to the late Oligocene and has a near global distribution. Today the genus is represented by a single species, the white shark *Carcharodon carcharias*. However, multiple extinct species are recognised in the Cenozoic including *Carcharodon hastalis*, *Carcharodon hubbelli* and *Carcharodon planus* comb. nov. Here we present the first occurrences of *Carcharodon hubbelli* and *Carcharodon planus* from New Zealand/Aotearoa, from the South Island/Te Waipounamu. *Carcharodon hubbelli* is previously reported from late Miocene deposits in the Pacific basin of North and South America, Asia, and Australia. Whereas *Carcharodon planus* has been reported from the Miocene of North America and Asia. One tooth from *Carcharodon hubbelli* is reported from the presumed late Miocene of Motunau Beach, North Canterbury, while one upper early Miocene tooth recovered from the Kakahu River, South Canterbury and an earliest Miocene tooth from the Big River mouth, Tasman District are referred to *Carcharodon planus*. In addition to being the first reported occurrences of these two species in New Zealand, the teeth of *Carcharodon planus* represent some of the earliest occurrences of the taxon as well as its southernmost occurrence.

## Introduction

The extant white shark, *Carcharodon carcharias* (Linnaeus 1758), is an iconic cosmopolitan apex predator in today’s oceans. Its fossil record extends back to the Pliocene (Ehret et al. 2012). Other species of *Carcharodon* have been identified in the fossil record and there is still much to learn about their geologic ages, distributions, and relationships (Ehret et al. 2009, 2012; Kent 2018). The most recently described species is *Carcharodon hubbelli* Ehret et al. 2012 from late Miocene (up to 5–7 mya) Pacific deposits of North and South America, Asia, and Australia (Ehret et al. 2012). *Carcharodon hubbelli* is a transitional evolutionary stage between *Carcharodon hastalis* (Agassiz 1843) and *C. carcharias* (Ehret et al. 2012). In older literature, the genus *Isurus* was attributed to the species *hastalis* and *planus*. While, the generic assignment of these closely-related species remains in flux (see Purdy et al. 2001; Ward and Bonavia 2001; Ehret et al. 2012; Kriwet et al. 2015; Yun 2022), for the time being, we follow Ehret et al. (2012) and Kent (2018) by considering it most parsimonious to place these shark species in the genus *Carcharodon*. *C. hastalis* had a global distribution and occurred from the late Oligocene to the lower Pliocene (Purdy et al. 2001; Kent 2018) whereas *Carcharodon planus* comb. nov. is only known with certainty from the Miocene of the Pacific (Boessenecker 2011; Nazarkin 2013; Yun 2022). Kemp (1991) reported Oligocene records of *C. planus* from Australia, though these records remain inconclusive because the teeth were not figured or described in any detail.

**Figure 1.**
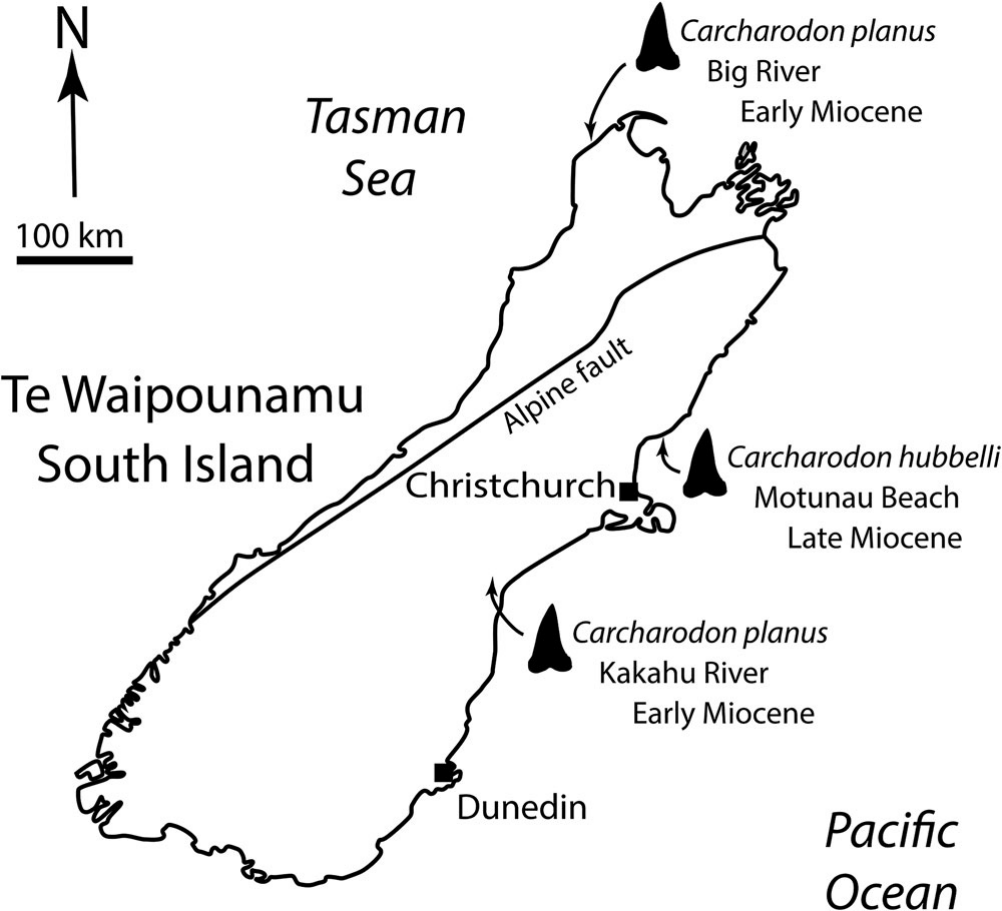
Localities for *Carcharodon hubbelli* and *Carcharodon planus* specimens from New Zealand.

To date, two species of *Carcharodon* have been reported from the fossil record of New Zealand/Aotearoa: *Carcharodon hastalis* from the Cenozoic (King et al. 2009) and *C. carcharias* from the Pliocene-Pleistocene (Keyes 1972). In this paper we document the first records of two other shark species from this clade: *Carcharodon planus* and *Carcharodon hubbelli*, both from Miocene deposits in the South Island/Te Waipounamu (Figure 1).

## Methods

Measurements of teeth were taken following Keyes (1972), Hubbell (1996), Shimada (2002a) and Ehret et al. (2009). Crown height (CH) is the vertical distance between a line, drawn across the lowest reaches where the tooth enameloid touches the root and the apex of the crown. Crown width (CW) was measured as the maximum transverse measurement across the crown where the crown enameloid meets the root. Crown thickness (CT) was measured in a medial position on the crown where thickness is greatest.

NMNZ = Museum of New Zealand Te Papa Tongarewa, Wellington, New Zealand.

## Results

Systematic paleontology

Class Chondrichthyes Huxley 1880

Order Lamniformes Berg 1958

Family Lamnidae Müller and Henle 1838

Genus *Carcharodon* Smith *in* Müller and Henle 1838

**Figure 2.**
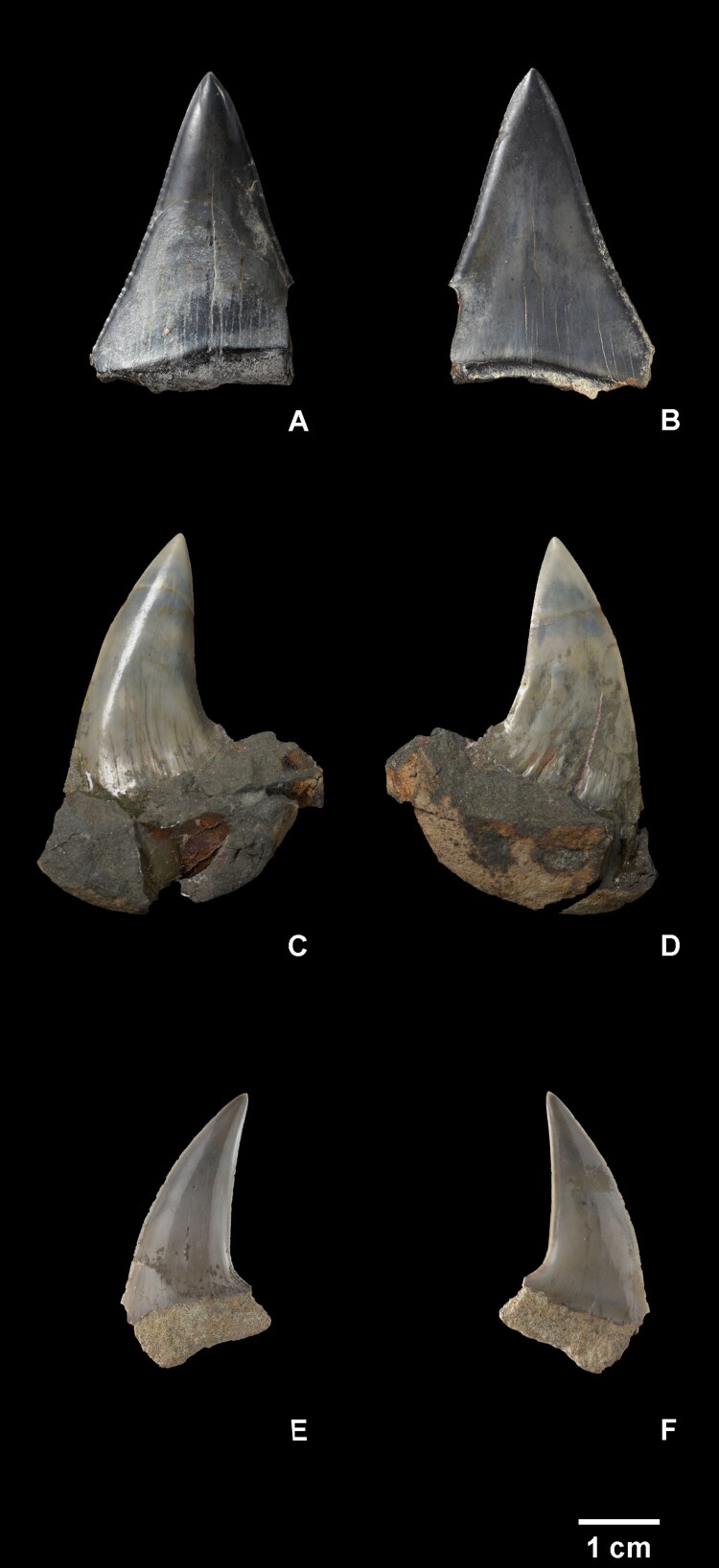
*Carcharodon hubbelli* (NMNZ S.48965) lingual (A), labial (B); *Carcharodon planus* (NMNZ S.44937) lingual (C), labial (D); *Carcharodon planus* (NMNZ S.49347) lingual (E), labial (F). Scale bar = 1 cm.

*Carcharodon hubbelli* Ehret et al. 2012 (Figure 2(A,B)).

**Description:** NMNZ S.48965 is a large triangular tooth with weakly serrated cutting edges, corresponding to an upper first or second anterior tooth. This tooth is approximately symmetrical and labiolingually compressed. A dental band (ie. chevron or bourlette) is not present, and the base of the crown is nearly straight both labially and lingually. Only a small fragment of the root remains. Fine serrations (up to 0.3 mm) are present along the basal ¾ of the crown and are largest basally; they become finer apically and the apical ¼ of the crown has a smooth cutting edge. The serrations are irregular in size and shape, occasionally with several smaller serrations in between larger. No notches between serrations are present. NMNZ S.48965 has a crown height of 38.6 mm, a crown width of >26.5 mm and a crown thickness of 8.8 mm.

**Identification:** Large, triangular, labiolingually compressed and flattened teeth lacking labiolingual curvature are assignable to the genus *Carcharodon*. Four named species (extant and extinct) of *Carcharodon* are currently recognised and three are considered chronospecies: *C. hastalis* for unserrated teeth (extinct), *C. hubbelli* for weakly serrated teeth (extinct), and *C. carcharias* for coarsely serrated teeth (extant). NMNZ S.48965 has weak, irregular serrations lacking notches, closely resembling teeth of *C. hubbelli*. Serrations are lower in height and less well defined than in previously reported teeth of *C. hubbelli* including the holotype (Ehret et al. 2012) and a tooth from the Purisima Formation of California (Boessenecker 2011, Figure 4). NMNZ S.48965 matches the description of *C. hubbelli* most closely, owing to the presence of weak serrations.

**Collection details:** Motunau Beach, North Canterbury, −43.049985, 173.074399. Collected by Morne Wium, 6 February 2021. Proximal end of crown found protruding from a small concretion, prepared out with pneumatic airscribe by M. Wium. New Zealand Fossil Record File Database (www.fred.org.nz) fossil record number N34/f0180 (Figure 1).

**Geological Setting:** The pholad-bored concretion of very fine sandstone that contained the tooth is referred to the Greta Formation. The fossiliferous concretions found on the wave cut platform beneath the sea cliffs near the Motunau River mouth derive from early Pleistocene (early Nukumaruan local stage) submarine canyon-head debris flow deposits that are transported, mixed and concentrated early Miocene to early Pleistocene (Otaian – Nukumaruan local stage; Aquitanian to Gelasian) fossiliferous clasts, exposed in the coastal cliffs (Lewis 1976; Beu and Maxwell 1990). The resulting issues of dating fossils found at Motunau beach are discussed by Fordyce (1991). The Greta Formation represents an upper bathyl setting, though the tooth-bearing concretion may originally derive from a continental shelf setting before being transported down submarine channels by debris flow (Browne and Field 1985; Beu and Maxwell 1990).

**Age:** The age presented for the tooth is inferred to be upper Tongaporutuan to Kapitean (NZ local stage; upper Tortonian – Messinian, Late Miocene). NMNZ S.48965 independently supports a late Miocene age for some of the reworked concretions in the early Pleistocene debris flow deposits of the Greta Formation, which is the source for the loose beach boulder lag at Motunau Beach. Feldmann and Keyes (1992) proposed that the numerous crustacean-containing concretions derived from the Greta Formation on Motunau beach were of upper middle to lower late Miocene age (Waiauan – Tongaporutuan local stage; Serravalian – Tortonian) due to the presence of *Tumidocarcinus giganteus* coupled with the constrains the depositional ages of debris flow clast sources presented by Lewis (1976). This information together with the reported age range for *C. hubbelli* elsewhere suggests the most plausible age range of initial deposition of NMNZ S.48965 to be upper Tongaporutuan to Kapitean (∼5.33–8.96 Ma; Raine et al. 2015). See more discussion below.

*Carcharodon planus* (Agassiz 1856)

**Description:** NMNZ S.44937 (Figure 2(C, D)) and S.49347 (Figure 2(E, F)) are large teeth with distally inclined crowns. The crowns are flattened and not labiolingually curved. In NMNZ S.44937 the mesial edge is sinuous, being mesially convex apically and concave basally; in NMNZ S.49347, the mesial edge is continuously convex, and despite breakage, appears to have been slightly mesially concave basally. The distal edge in NMNZ S.44937 is sinuous, being slightly convex apically and strongly concave basally; in NMNZ S.49347 the distal edge is nearly straight. In both teeth, the apex of the crown is positioned distally to the distal crown heel. The crown is labiolingually compressed and narrow, with a flat labial face; in NMNZ S.44937 the labial face exhibits several longitudinal wrinkles along the basal 5.0 mm of the crown. The root is broken in NMNZ S.49347 and in NMNZ S.44937 it is obscured by a nodule. In NMNZ S.49347 damage precludes determination of the shape, but the root seems to have been apicobasally shallow and likely had rectangular proportions – though whether the lobes were rounded is unclear. NMNZ S.44937 has a crown height 0f 30.2 mm, a crown width of ∼22 mm and a crown thickness of 8.3 mm. NMNZ S.49347 has a crown height of 26.9 mm, a crown width of >16.3 mm and a crown thickness of 7.0 mm.

**Identification:** NMNZ S.44937 and S.49347 are clearly large lamnid shark teeth owing to the triangular shape, roughly rectangular root, and labiolingual compression. The strong distal inclination, strongly convex (rather than straight) to even sinuous mesial edge of the crown is shared only with *Carcharodon planus*. Upper lateral teeth of ancestral *C. hastalis* can be distally inclined, however these teeth are typically always triangular with approximately straight mesial and distal cutting edges. The specimens discussed here have a much more pronounced distal inclination than any teeth of *C*. *hastalis* seen by any of the authors. Although the roots are obscured or broken in both specimens, we feel confident in the identifications due to the shape of the crowns. Previous authors have suggested that *Carcharodon planus* exhibits a triangular root with rounded lobes, which is shared with other closely related species and is not itself a diagnostic characteristic (Kuga 1985; Karasawa 1989; Nazarkin 2013; Yun 2022). NMNZ S.44937 was initially identified as *Carcharodon hastalis*, then as *Parotodus* in the lab, but we note that this specimen differs considerably from *Parotodus* by being linguo-labially flattened, bearing sinuous mesial and distal margins, and by lacking the distinctive dental chevron (or bourlette) on the lingual side of the tooth that distinguishes all Otodontidae.

**Taxonomic Note:** We provisionally refer the species *Cosmopolitodus planus* (or alternatively *Isurus planus*) to the genus *Carcharodon* and recombine it as *Carcharodon planus*. *Carcharodon planus* is distinctive in possessing laterally recurved teeth and rounded root lobes but otherwise is quite similar to *Carcharodon hastalis* (Jordan and Hannibal 1923): broad, labiolingually flattened crown, unserrated cutting edges, and labiolingually flattened and subrectangular root. Amongst Neogene and extant species of lamnid sharks, it shares these features in common only with *Carcharodon hastalis,* and aside from serrations, *Carcharodon hubbelli* and *Carcharodon carcharias* as well. Similarities with *Carcharodon hastalis* led to initial assignment as *Isurus* and *Cosmopolitodus* when *C. hastalis* was formerly assigned to these genera (Purdy et al. 2001; Nazarkin 2013; Yun 2022). We consider that *C. planus* is congeneric with *C. hastalis*, the latter of which was assigned to *Carcharodon* by Ehret et al. (2012). We follow Ehret et al. (2012) here but note that others have assigned both to *Cosmopolitodus* (Yun 2022). Despite being common in Pacific rim Miocene marine strata, *Carcharodon planus* is poorly studied, and we recommend that future investigations utilise morphometric or other quantitative methods to assess the taxonomy of this species (e.g. Nyberg et al. 2006; Türtscher et al. 2021).

Referred Specimen NMNZ S.44937 (Figure 2(C,D))

**Collection Details:** Kakahu River, 2.5 km west of Hilton, South Canterbury, −44.137334, 171.136506. Collected by Graeme M. Mason, 1972 (field number FM7), in situ from siltstone. M. Richards’ field observations (5 April 2021) suggest that the specimen was collected from the base of the riverbank cliffs on the true left bank ∼70 m upstream of the provided coordinates in the New Zealand Fossil Record File Database (www.fred.org.nz) fossil record number J38/f0002.

**Geological Setting:** Collected from a massive blue-grey siltstone of the Mount Harris Formation ( = Tokama Siltstone of Field and Browne 1986). Chemically weathered outcrops are buff yellow. The Mount Harris Formation was deposited on a wide continental shelf of probable outer shelf depth (Beu and Maxwell 1990) (Fig 1).

**Age:** Inferred to be upper early Miocene (Altonian local stage; 15.9-18.7 Ma) via stratigraphic placement high in the formation by GM Mason. A fossil dolphin collected locally from the same formation, though possibly a lower part of the sequence (J38/f0113), yielded an Altonian assemblage of foraminiferans (Aguirre-Fernández 2013).

**Referred Specimen NMNZ S.49347** (Figure 2(E,F))

**Collection Details:** At prominent rock outcrop ∼120 m elevation on hilltop of eastern side of Big River outlet, Tai Tapu Station, Northwest Nelson area, Tasman District, −40.766079, 172.264060. Collected in situ by shepherd Leonard Mason, March 2021, donated to NMNZ by Piers Maclaren at the request of landowners Dave and Elva Harwood. Field photos by L. Mason show that the tooth root was not preserved in the rock, possibly being bioeroded during deposition. New Zealand Fossil Record File Database (www.fred.org.nz) fossil record number L25/f0019 (Figure 1).

**Geological Setting:** Collected from the Kaipuke Siltstone, a grey indurated massive calcareous siltstone that was deposited in a mid-shelf setting in water temperatures ranging between ∼12-17°C (Aguirre-Fernández and Fordyce 2014).

**Age:** Inferred to be earliest Miocene (upper Waitakain local stage; Aquitanaian). Fossil odontocete *Papahu taitapu* (OU 22066; M25/f0057) was collected from the Kaipuke Siltstone at a shore platform ∼15 km northeast of NMNZ S.49347. The cetacean was stratigraphically ∼10 m upsequence from the conformable contact with the underlying Takaka Limestone (Aguirre-Fernández and Fordyce 2014), and was originally dated by a foraminiferal fauna sampled along strike (M25/f0059) to early Miocene (upper Waitakian – Otaian; Aquitanian – mid Burdigalian). New strontium isotope data by Marx et al. (in press) from *Lentipecten* shells associated with OU 22066 produce a weighted mean Sr ratio of 0.708271 ± 0.000027 (2se) giving an age of 22.73 ± 0.53 Ma. This refines the lower Kaipuke Siltstone to the upper Waitakian (Aquitanian). The shark tooth locality is inferred to be of similar age due to its stratigraphic placement close to the underlying Takaka Limestone.

## Discussion

We document records of two poorly known large mackerel shark species from the New Zealand fossil record. *Carcharodon planus* was previously known from the lower through upper Miocene rocks (Burdigalian through Tortonian) of the Pacific Rim, particularly from the west coast of the United States (Jordan and Hannibal 1923; Boessenecker 2011) and Mexico (Deméré 1984), Australia (Kemp 1991), the Korean Peninsula (Yun 2022), Sakhalin Island (Nazarkin 2013), and Japan (Kuga 1985; Karasawa 1989). The New Zealand records extend the range of this species further southwest in the Pacific basin however this is unsurprising given the Australian occurrences. At 44° S, this new record of *Carcharodon planus* from New Zealand is the highest latitude occurrence of this taxon in the Pacific basin, paralleling the record in the western North Pacific (47° N, Sakhalin; Nazarkin 2013), and further indicating a wide latitudinal range for this species. Curiously, teeth of *C. planus* have not been reported from the densely sampled and rich fossil beds of Peru and Chile (e.g. Chávez-Hoffmeister and Villafaña 2023), suggesting a circum-North Pacific and western South Pacific distribution.

NMNZ S.44937 and NMNZ S.49347 represent early Miocene records of *Carcharodon planus*. A similar-aged record of *Carcharodon planus* from the Astoria Formation of Oregon (USA) was listed (but not figured) by Welton (1972); this unit dates to 17.3-16.6 Ma according to paleomagnetism (Prothero et al. 2001). The youngest known occurrence of *Carcharodon planus* appears to be the Santa Margarita Sandstone of central California (Perry 1993), estimated to be 10–9 Ma in age (Repenning and Tedford 1977). Accordingly, the species is common and conspicuous in this stratum and the Sharktooth Hill Bonebed in the Langhian Round Mountain Silt of California (Boessenecker, pers. obs.) but absent from well-sampled (albeit poorly documented) marine vertebrate assemblages from the ‘late’ Tortonian upper Monterey Formation (Barnes et al. 1985: Table 1) and the lower San Mateo Formation (Barnes et al. 1981) of southern California. Notably, teeth of *Carcharodon hastalis* and *Isurus oxyrinchus* are common in these assemblages, suggesting an extinction likely during the Tortonian, perhaps 8–10 Ma.

*Carcharodon hubbelli* also appears to be a Pacific basin endemic species, being reported from Peru (Muizon and DeVries 1985; Ehret et al. 2009, 2012), California (Boessenecker 2011), Japan (as *Carcharodon* sp.; Yabe 2000), and Australia (Kemp 1991, Fig. 19 h). The typical range of dates for specimens of this taxon from well-dated deposits is latest Miocene, approximately 5–7 Ma (Boessenecker 2011; Ehret et al. 2012; Ochoa et al. 2022). The New Zealand record therefore also extends the range of this species further to the southwest in the Pacific. The age of fossils from the boulder lag of concretions found on Motunau Beach, North Canterbury, is difficult to determine, however *C. hubbelli* can be used as a high-resolution biostratigraphic indicator due to its well constrained age range (Ehret et al. 2012). A latest Miocene age (Kapitean local stage; Messinian) for NMNZ S.48965 is likely as the Kapitean (5.33-7.2 Ma; Raine et al. 2015) overlaps nicely with the ages of overseas records. An upper Tongaporutuan age (∼7.2–8.96 Ma; Raine et al. 2015), at the maximum end of *C. hubbelli*'s known age range, is possible for NMNZ S.48965 because it has the least-developed serrations yet recorded for this transitional species. Also, a Kapitean age has yet to be reported for any fossiliferous concretionary material at Motunau whilst some macrofossils previously described at the site have proposed Tongaporutuan or older ages (Lewis, 1976; Feldmann and Keyes 1992). However, macrofossils such as mollusca can be hard at times to distinguish between these two New Zealand stages (Beu & Maxwell 1990), so a conservative approach of late Miocene (upper Tongaporutuan – Kapitean; upper Tortonian – Messinian) is preferred. Further work is required to date the fossiliferous concretion assemblage exposed on the Motunau coastline, though temporally constrained specimens like *C*. *hubbelli* aid in refining the ages for some of the ancient debris-flows’ source stratigraphy.

*Carcharodon planus* seems to have been more modest in size relative to extant *Carcharodon carcharias*. Large teeth of *C. planus* from the Round Mountain Silt of California on display at CCNHM (Mace Brown Museum of Natural History, Charleston, South Carolina, USA) measure up to 31 mm in crown height, corresponding to a body length of 3.2 metres (based on tooth:body length regressions of *Isurus oxyrinchus*; Shimada 2002b) to 3.6 metres (based on tooth:body length regressions of *Carcharodon carcharias*; Shimada 2002a). Whereas Ehret et al. (2009) and Ehret et al. (2012) showed that *C. hubbelli* grew at a slower rate than modern *C. carcharias* but reached similar sizes. The addition of these two sharks to the New Zealand Miocene fauna demonstrates that the Cenozoic seas of New Zealand had a more diverse fauna of apex-predators than previously recorded.
